# A novel splicing variant of *ANXA11* in a patient with amyotrophic lateral sclerosis: histologic and biochemical features

**DOI:** 10.1186/s40478-021-01202-w

**Published:** 2021-06-07

**Authors:** Makoto Sainouchi, Yuya Hatano, Mari Tada, Tomohiko Ishihara, Shoichiro Ando, Taisuke Kato, Jun Tokunaga, Gaku Ito, Hiroaki Miyahara, Yasuko Toyoshima, Akio Yokoseki, Tetsutaro Ozawa, Kohei Akazawa, Osamu Onodera, Akiyoshi Kakita

**Affiliations:** 1 Departments of Pathology, Brain Research Institute, Niigata University, 1-757 Asahimachi, Chuo-ku, Niigata, 951-8585 Japan; 2 Departments of Neurology, Brain Research Institute, Niigata University, 1-757 Asahimachi, Chuo-ku, Niigata, 951-8585 Japan; 3 Department of System Pathology for Neurological Disorders, Brain Science Branch, Brain Research Institute, Niigata University, Niigata, Japan; 4 Department of Neurology, Uonuma Institute of Community Medicine, Niigata University Medical and Dental Hospital, 4132 Urasa, Minamiuonuma, Niigata 949-7302 Japan; 5 Department of Medical Informatics, Niigata University Medical and Dental Hospital, 1-754 Asahimachi, Chuo-ku, Niigata, 951-8520 Japan

## To the editor

Mutations in the annexin A11 gene (*ANXA11)* have been shown to cause amyotrophic lateral sclerosis (ALS) [6]. Annexin A11 is a Ca^2+^-dependent phospholipid-binding protein that possesses an N-terminal low-complexity domain and C-terminal repeated annexin domains, being involved in Ca^2+^ signaling, cell division, apoptosis, and vesicle trafficking [3, 5, 8]. Recent studies have indicated that ALS-related *ANXA11* mutations enhance aggregation propensity, leading to dysregulation of intracellular Ca^2+^ homeostasis and RNA granule transport [3, 5, 6].

The clinical phenotypes of *ANXA11*-mutated ALS vary, and differ even in patients harboring the same mutation [6, 7, 9]. Autopsy findings of two patients harboring N-terminal *ANXA11* mutation have been reported. One patient with p.D40G *ANXA11* mutation showed the features of classical ALS [6], while the other with p.G38R *ANXA11* mutation showed those of ALS-TDP with frontotemporal lobar degeneration (FTLD)-TDP type A [4, 7]. Both patients showed aggregation of 43 kDa TAR-DNA-binding protein (TDP-43) and annexin A11 in neurons (Table 2). However, the variety of neuropathologic features, including the distribution and morphology of TDP-43- or annexin A11-immunoreactive (ir) inclusions, in patients with *ANXA11* mutations remain to be further clarified. Here, we investigated the clinicopathologic features of a Japanese patient with sporadic ALS harboring a novel splicing mutation in the annexin domain of *ANXA11*, and the functional significance of the mutation.

A 57-year-old man, who had no family history of neurologic disorders, presented with progressive limb weakness and stiffness, followed by muscle fasciculations, pyramidal signs, dysphagia and dysarthria. No dementia was noted. He died suddenly 19 months after disease onset. His detailed clinical features are described in the Additional File.

**Fig. 1:**
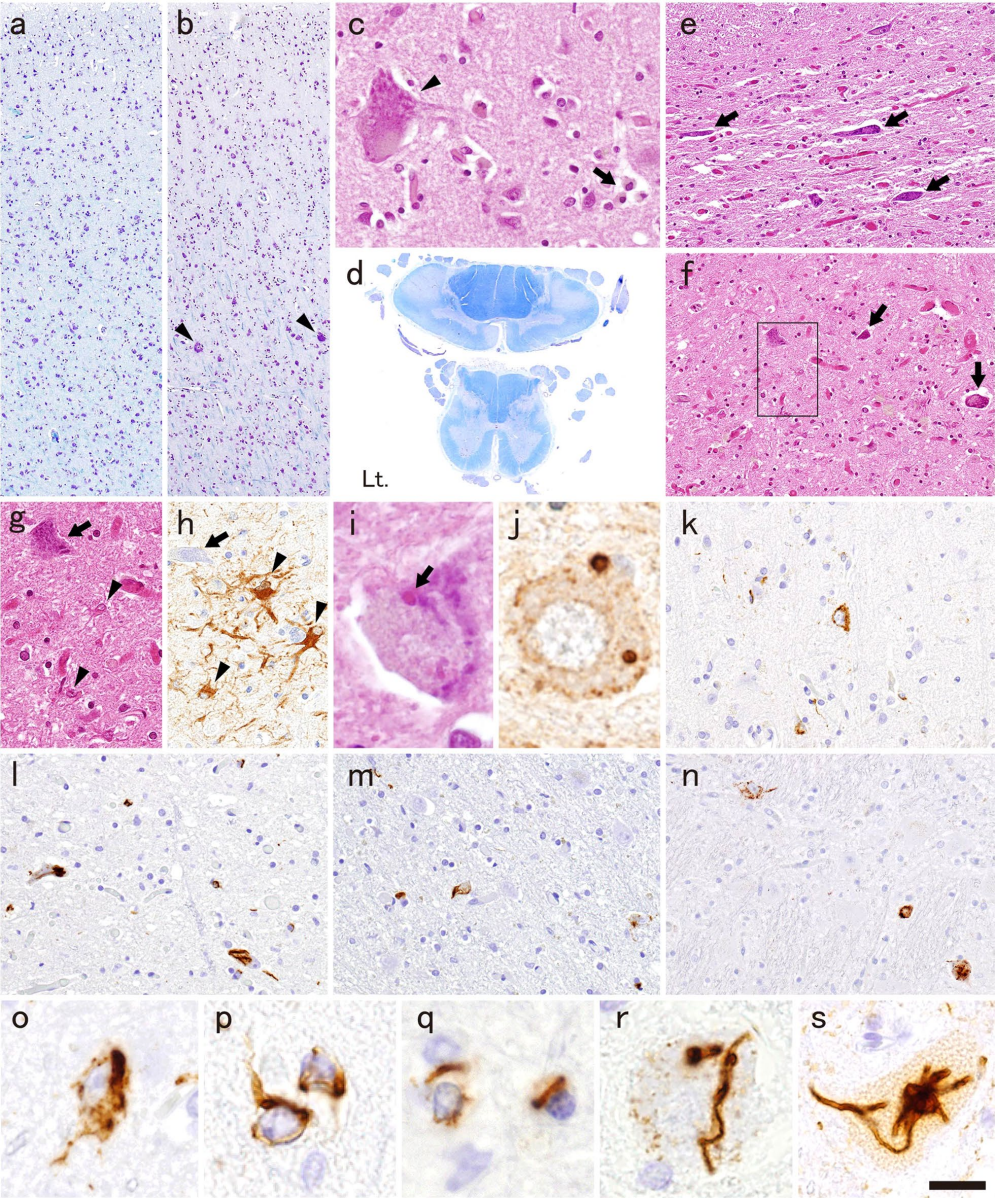
Neuropathologic findings. **a, b** The motor cortex shows neuronal loss and gliosis more prominently in **a** the lateral side than **b** the medial side. Some Betz cells are preserved only in the medial side of the motor cortex (arrowheads in **b**, **c**). **c** Macrophage accumulation in a Betz cell-sized hole (arrow in **c**), indicating ongoing neuronal degeneration, is evident in the medial side. **d** Myelin pallor in the bilateral lateral columns of the cervical and lumbar cord, and the left anterior column of the cervical cord, which indicate degeneration of the pyramidal tracts. Atrophy of the anterior horns is also evident in the cervical cord. **e**–**h** Severe neuronal loss and gliosis in **e** the cervical anterior horn, and **f**–**h** facial nucleus (arrows, atrophic neurons; arrowheads, reactive astrocytes; **g** and **h**, high magnification images of the square in **f**; **h**, GFAP-immunohistochemistry). **i**, **j** Bunina bodies in the remaining motor neurons of the facial nucleus (**j**, cystatin C-immunohistochemistry). **k**–**s** Phosphorylated TDP-43 (pTDP-43)-immunohistochemistry. Several pTDP-43-immunoreactive (ir) neuronal cytoplasmic inclusions (NCIs) and glial cytoplasmic inclusions (GCIs) in **k** the motor cortex, **l** hypoglossal nucleus and **m** subthalamic nucleus. **n** NCIs in the pontine nucleus. **o** Granulofilamentous and (left in **p**) filamentous NCIs and (right in **p**) GCI in the motor cortex. **q** GCIs in the white matter adjacent to the motor cortex. **r** Thick skein-like NCI in the hypoglossal nucleus. (**s**) Tube-shaped NCI in the anterior horn of the lumbar cord. Bar = 190 µm for **a**, **b**; 35 µm for **c**, **g**, **h**; 3 mm for **d**; 75 µm for **e**, **f**; 10 µm for **i**, **j**, **o**–**r**; 50 µm for **k**–**n**; 15 µm for **s**

The neuropathologic features were consistent with ALS. Neuronal loss and gliosis were restricted to the motor cortex, brainstem motor nuclei, and spinal anterior horns (Fig. 1a–c, e–h; Table 1). The corticospinal tract in the spinal cord showed severe degeneration (Fig. 1d). Bunina bodies were occasionally found in the lower motor neurons (Fig. 1i, j). Phosphorylated TDP-43 (pTDP-43)-ir neuronal and oligodendroglial cytoplasmic inclusions (NCIs and GCIs, respectively) were evident in the above-mentioned areas (Fig. 1k, o, p, motor cortex; q, white matter adjacent to the motor cortex; l, r, hypoglossal nucleus; s, anterior horn of the lumbar cord) and several others (Fig. 1m, subthalamic nucleus; n, pontine nucleus) but not in the hippocampus or temporal cortex (Table 1). Morphologically, the pTDP-43-ir NCIs were granular and/or filamentous (Fig. 1o, p) in all areas where NCIs were observed, and characteristically those in the lower motor neurons were thick skein-like, or tube-shaped (Fig. 1r, s).

**Table 1:** Distribution of neuronal loss and pTDP-43- and annexin A11-immunoreactive NCIs and GCIs

	Neuronal loss	pTDP-43		Annexin A11	
		NCIs	GCIs	NCIs	GCIs
**Cerebrum**					
Frontal cortex	−	−	−	−	−
Motor cortex	++	++	++	+	−
White matter*	+**	NA	++	NA	−
Temporal cortex	−	−	−	−	−
Parietal cortex	−	+	−	−	−
Occipital cortex	−	−	−	−	−
**Subcortical area**					
Ammon/dentate gyrus	−	−	−	−	−
Amygdaloid nucleus	−	−	−	−	−
Caudate nucleus/putamen	−/−	+/+	−/+	−/+	−/+
Globus pallidus	−	−	−	−	−
Thalamus	−	−	−	−	−
Subthalamic nucleus	−	+	+	−	−
**Brainstem**					
Oculomotor nucleus	−	−	+	−	−
Substantia nigra	−	−	−	−	−
Locus ceruleus	−	+	−	−	−
Trigeminal motor nucleus	+	+	−	+	−
Facial nucleus	++	++	+	++	−
Pontine nucleus	−	+	+	+	−
Ambiguous nucleus	+	+	−	−	−
Hypoglossal nucleus	++	++	++	++	−
Inferior olivary nucleus	−	+	+	+	−
Reticular formation	−	+	+	+	−
**Cerebellum**					
Cortex/white matter	−/−**	−/NA	−/+	−/NA	−/−
Dentate nucleus	−	−	−	−	−
**Spinal cord**					
Anterior horn (C/Th/L)	++/++/+	+/+/++	++/++/++	+/+/++	−/−/+
Lateral funiculus (C/Th/L)	++/++/+**	NA	−/+/+	NA	−/−/−
Anterior funiculus (C/Th/L)	++/++/−**	NA	+/+/+	NA	−/−/−

*, White matter adjacent to the motor cortex; Neuronal loss (**, degeneration with macrophage infiltration): −, none; +, mild to moderate; ++, severe; NCIs and GCIs: −, none; +, rare (1–4 /10 high-power fields); ++, occasional (≧ 5/10 high-power fields); pTDP-43, phosphorylated 43 kDa TAR DNA-binding protein; NCIs, neuronal cytoplasmic inclusions; GCIs, glial cytoplasmic inclusions; NA, not applicable; C, cervical cord; Th, thoracic cord; L, lumbar cord

**Fig. 2:**
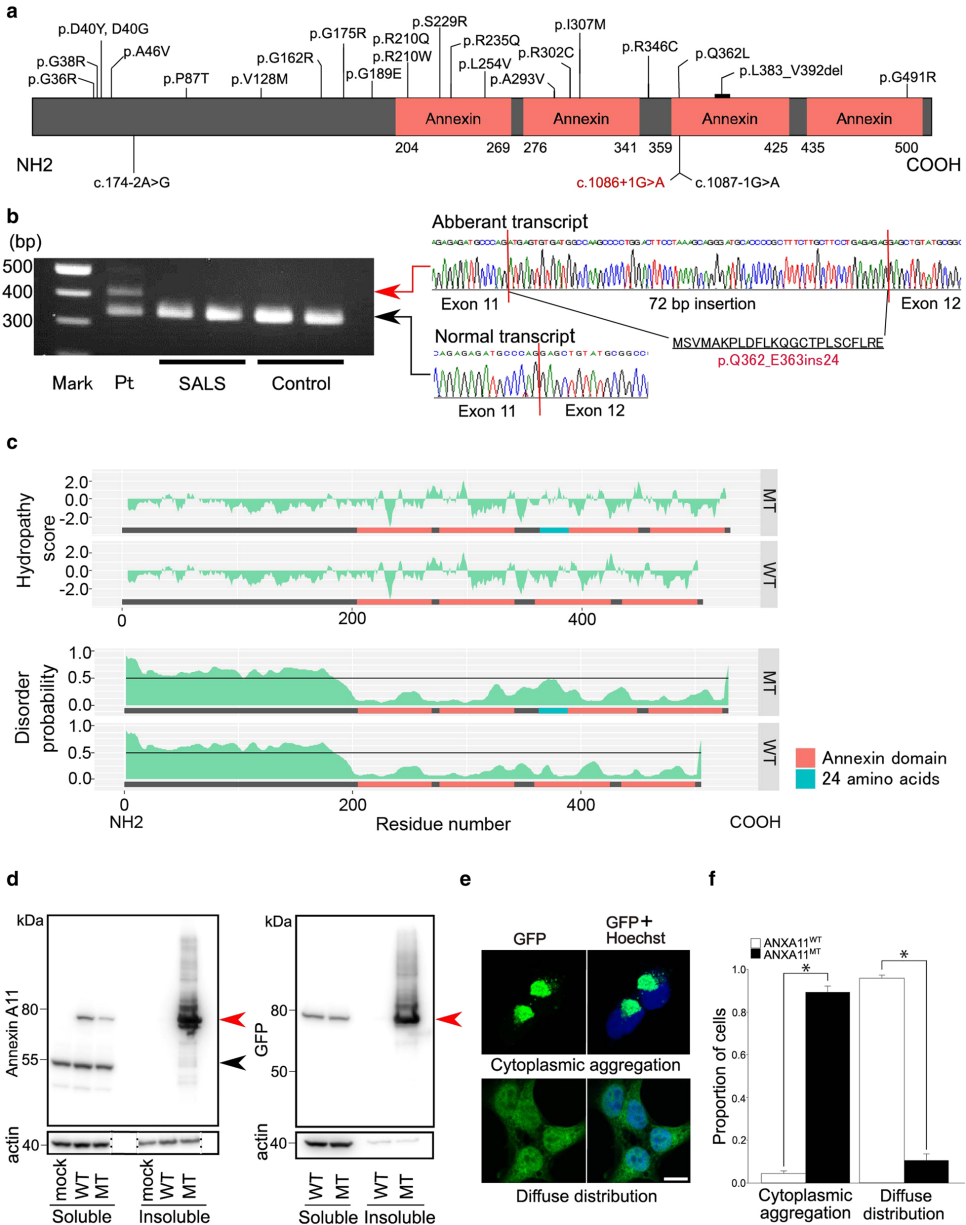
Position of the identified *ANXA11* mutation and biological characteristics of the mutant annexin A11. **a** Annexin A11 protein domains and the position of mutations identified in patients with amyotrophic lateral sclerosis (ALS). Mutations found in the previous studies are in black and that of the present patient is in red. **b** An aberrant *ANXA11* mRNA splicing as a consequence of the c.1086 + 1G > A mutation. Gel electrophoresis shows the reverse transcription PCR products from the autopsied brain of the present patient (Pt), patients with sporadic ALS harboring no *ANXA11* mutations (SALS), and controls. The lane of Pt contains two different bands. Sequencing results show that the band located at 339 bp implies a normal transcript, and that located at 411 bp implies an aberrant transcript caused by 72-bp insertion between exons 11 and 12, numbered on the basis of ENSEMBL ANXA11-203. The inserted sequence results in a 24-amino-acid insertion. **c** Hydrophobicity and protein disorder predictions. Upper panel: Hydropathy plot [2] of the aberrant annexin A11 (MT) obtained using ExPASy shows increased hydrophobicity around the inserted 24-amino-acid site. Lower panel: Disorder profile plot of the aberrant annexin A11 (MT) obtained with PrDOS [1] shows increased disorder probability around the inserted 24-amino-acid site compared with that of the wild type (WT), indicating that the aberrant splicing could affect the stability and function of annexin A11. **d** Solubility fractionation of the cells transfected with GFP-tagged ANXA11 (GFP-ANXA11) constructs, followed by Western blotting using antibodies for (left) annexin A11 and (right) GFP. The wild type annexin A11 protein is predominantly soluble, whereas the mutant construct shows a prominent increase in the insoluble fraction. Red arrowhead: wild type (WT) and mutant (MT) GFP-annexin A11. Black arrowhead: endogenous annexin A11. β-Actin: loading control. **e** GFP-annexin A11 localization in HEK293T cells. Cells are classified according to GFP-annexin A11 localization patterns, i.e. cytoplasmic aggregation, and diffuse distribution. Bar = 10 µm for all images in **e**. **f** Proportion of cells showing each GFP-annexin A11 localization pattern. The proportion of cells showing cytoplasmic aggregation is significantly higher, and that of cells showing diffuse distribution is significantly lower in cells expressing GFP-annexin A11^MT^ than in those expressing GFP-annexin A11^WT^ (*, *p* < 0.002; Mann–Whitney *U* test)

Genetic screening of ALS-related mutations through whole-exome sequencing revealed a heterogeneous splice site mutation, c. 1086+1G>A, in the *ANXA11* gene (Fig. 2a), which has an allele frequency of 0.08% in the Human Genetic Variation Database (HGVD), and has not been documented in the Exome Aggregation Consortium (ExAC). On the other hand, no variants which are less than 0.1% frequency were found in any of the other ALS-related genes (See Supplementary methods). A splice site prediction program (NetGene2, http://www.cbs.dtu.dk/services/NetGene2/) correctly recognized the splice donor site of intron 11 in the wild type, but not in the mutant sequence, indicating that the mutation disrupts the splice donor site. Sequencing of the reverse transcription PCR products of *ANXA11* mRNA obtained from the autopsied brain showed an aberrant transcript containing a 72-bp insertion between exons 11 and 12 that resulted in the insertion of 24 amino acids (Fig. 2b). Using the structure predictive tool, PSIPRED 4.0 (http://bioinf.cs.ucl.ac.uk/psipred/), the inserted amino acid sequence was predicted to create new alpha helixes. However, no new motifs were identified in the aberrant protein using a motif finder (Pfam 34.0, http://pfam.xfam.org/ and HMMER v3.3.2, http://hmmer.org/). Hydrophobicity prediction [2] using ExPASy (https://web.expasy.org/protscale/) and protein disorder analysis using PrDOS (http://prdos.hgc.jp/cgi-bin/top.cgi) [1] revealed that the aberrant annexin A11 had increased hydrophobicity (Fig. 2c, upper panel) and disorder probability (Fig. 2c, lower panel), respectively, around the inserted 24-amino-acid site compared with wild type. We then undertook cellular experiments using HEK293T cells transfected with the 72-bp inserted mutant, and wild type GFP-tagged ANXA11 (GFP-ANXA11^MT^ and GFP-ANXA11^WT^, respectively) constructs. Solubility fractionation of the cells transfected with GFP-ANXA11 constructs, followed by Western blotting using antibodies for annexin A11 (Fig. 2d, left) and GFP (Fig. 2d, right) revealed that GFP-annexin A11^MT^ formed detergent-resistant insoluble species. Furthermore, cells expressing GFP-annexin A11^WT^ showed mainly a diffuse nuclear and cytoplasmic distribution of GFP, whereas cells expressing GFP-annexin A11^MT^ showed cytoplasmic aggregation significantly frequently (Fig. 2e, f).

**Fig. 3:**
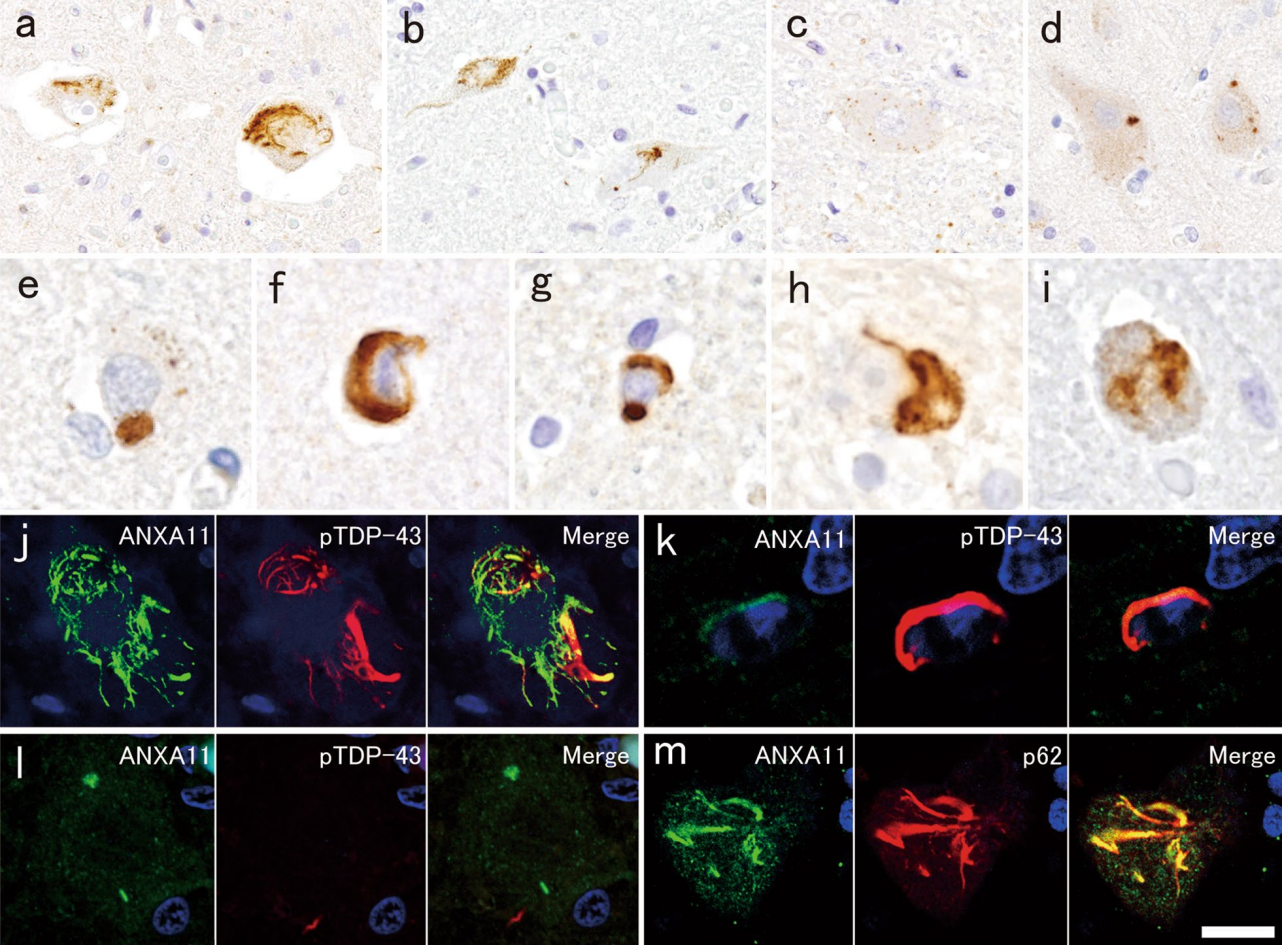
Expression of annexin A11 in autopsied CNS tissue. **a**–**i** Annexin A11-immunohistochemistry. **a** Annexin A11-immunoreactive (ir) skein-like neuronal cytoplasmic inclusions (NCIs) in the anterior horn and **b** hypoglossal nucleus. **c** Small dot-like staining pattern in an anterior horn cell from a patient with amyotrophic lateral sclerosis without *ANXA11* mutation. **d** Small irregular-shaped and **e** round NCIs in the motor cortex. **f** Filamentous NCI and **g** glial cytoplasmic inclusion (GCI) in the putamen. **h** Filamentous NCI in the pontine nucleus and **i** inferior olivary nucleus. **j**–**l** Double-label immunofluorescence with annexin A11 and pTDP-43. **j** Annexin A11 and pTDP-43 are partially co-localized in the skein-like NCI. **k** Weak immunoreactivity of annexin A11 in pTDP-43-ir GCI. **j**, **k**, lumbar anterior horn. **l** Annexin A11-ir and pTDP-43-negative small, irregularly shaped and short linear NCIs, and pTDP-43-ir and annexin A11-negative dystrophic neurite in the motor cortex. **m** Annexin A11-ir and p62-ir NCI in the lumbar anterior horn. **j**–**l** green, annexin A11; red, pTDP-43, **m** green, annexin A11; red, p62. Bar = 60 µm for **a**, **b**; 30 µm for **c**, **d**; 10 µm for **e**–**i**, **l**; 15 µm for **j**, **m**; 6 µm for **k**

Indeed, the present patient had many annexin A11-ir skein-like NCIs in the lower motor neurons (Fig. 3a, b). In contrast, sparse dot-like cytoplasmic staining (Fig. 3c) and rare filamentous inclusions were observed in the lower motor neurons of ALS patients with no *ANXA11* mutation. Such sparse dot-like cytoplasmic staining was also observed in controls, and not only in motor neurons (Additional file 1: Fig. 1). These findings indicated that annexin A11-ir NCIs are not completely specific, but characteristic to *ANXA11*-mutated patients. In the motor cortex, only a few, irregularly shaped or round, small NCIs were observed (Fig. 3d, e). Filamentous NCIs were also evident in several regions where pTDP-43-ir NCIs were also present (Fig. 3f, h, i, Table 1). Annexin A11 aggregated preferentially in neurons, although a few GCIs were observed (Fig. 3g). Double-labeling immunofluorescence for pTDP-43 and annexin A11 revealed that annexin A11 and pTDP-43 were partially co-localized in skein-like NCIs, and a few GCIs in the anterior horns (Fig. 3j, k), but not in small annexin A11-ir NCIs in the motor cortex (Fig. 3l). Annexin A11-ir skein-like NCIs in the anterior horns were frequently well labeled for p62 (Fig. 3m). Details of methods are in Supplementary methods, and the primary antibodies used are listed in Supplementary table 1 in Additional file 1.

The neuropathologic features of this patient were indistinguishable from those of ALS without known mutations, except for the presence of annexin A11-ir NCIs, being similar to the previously reported patient with *ANXA11* mutation (Table 2) [6].

**Table 2:** Clinical and neuropathologic features of patients harboring *ANXA11* mutation

	Present patient	Patient with p.D40G [6]	Patient with p.G38R [7]
**Clinical features**			
Age at onset (years)	57	72	60’s^a^
Disease duration (months)	19	36	na^a^
Initial symptom	Limb	Bulbar	Limb
Cognitive impairment or FTD	−	−	+
**Neuropathologic phenotype**	ALS-TDP	ALS-TDP	ALS-TDP with FTLD-TDP type A
**Neuronal loss**			
Motor cortex	+	na	+
Lower motor neurons	+	+	na
Frontotemporal cortex	−	na	+
Degeneration of CST	+	+	+
**Bunina body**	+	na	+
**pTDP-43-ir NCIs/GCIs**	+/+	+/na	+/+
**Annexin A11-ir NCIs/GCIs**	+/few	+/na	+/na
Morphology of annexin A11-ir NCIs	Skein-like, filamentous, small round	Skein-like, filamentous, large-caliber and tube-shaped, large basket-like	Tube-shaped, large conglomerate, large round
Co-localization of pTDP-43 and annexin A11	− or +	−	− or +

+, present; −, absent; a, among 3 patients harboring p.G38R *ANXA11* mutation, the autopsied patient was not identified in the reference [7]; FTD, frontotemporal dementia; ALS, amyotrophic lateral sclerosis; FTLD, frontotemporal lobar degeneration; na, not available; CST; corticospinal tract; pTDP-43, phosphorylated 43 kDa TAR DNA-binding protein; ir, immunoreactive

On the other hand, genetic screening revealed the novel splicing mutation in the C-terminal of *ANXA11*, and the in silico analysis and cellular experimental findings indicated that the aberrantly spliced transcript induced cytoplasmic accumulation and enhanced the aggregation propensity of annexin A11, suggesting that the mutation had pathogenicity. Functional studies of ALS-related *ANXA11* mutations have shown that both N-terminal and C-terminal mutations induced abnormal phase separation to form aggregates, leading to the functional defects of annexin A11 [3, 5]. Similar toxic-gain-of-function mechanisms might have contributed to ALS pathogenesis in this patient.

Morphologically, the annexin A11-ir NCIs in this patient differed slightly from those in the previous patients with N-terminal *ANXA11* mutation [6, 7]. We observed skein-like, filamentous, and small round inclusions but not the conglomerated, round, or basket-like large inclusions that had been present in the previous patients. Such differences might depend on the specific location of each mutation, since the disorder probability is lower in the annexin domain than in the N-terminal low complexity domain (Fig. 2c), which is responsible for protein aggregation [5]. Furthermore, the co-localization of annexin A11 and p62 in NCIs was clear in this patient, but not in the previous patient with N-terminal *ANXA11* mutation [7], implying that clear and frequent superposition of annexin A11 and p62 might be specific for the patients with C-terminal *ANXA11* mutation.

Interestingly, annexin A11 was aggregated predominantly in neurons and only very sparsely in glial cells, and topographically, in the brainstem motor nuclei and spinal anterior horns rather than the motor cortex, whereas TDP-43 was aggregated in both neurons and glial cells, and frequently in both the upper and lower motor systems. Thus, TDP-43 aggregated even in annexin A11-negative cells, especially glial cells. Similarly, TDP-43 aggregation in annexin A11-negative cells had also been noted in the two previously reported patients with N-terminal *ANXA11* mutation [6, 7]. Together with these previous studies, our present findings indicate that annexin A11 aggregation propensity is probably dependent on cell type, and that annexin A11 aggregation is not indispensable for triggering TDP-43 aggregation and neurodegeneration.

Overall, we have confirmed the pathogenicity of this novel mutation in the C-terminal region of *ANXA11*. Neuropathologic findings suggested a cell type-dependent annexin A11 aggregation propensity. Further studies are needed to confirm this possibility.

## Supplementary Information

**Additional file 1.** Details of methods and case presentation, a list of the primary antibodies used (Supplementary Table 1), and the representative images of annexin A11 immunohistochemistry in CNS tissue of controls (Supplementary Figure 1).

## Data Availability

The datasets used and analyzed during the current study available from the corresponding author on reasonable request.

## Abbreviations

ALS: Amyotrophic lateral sclerosis
pTDP-43: Phosphorylated 43 kDa TAR-DNA-binding protein
