# Correction to: A novel splicing variant of *ANXA11* in a patient with amyotrophic lateral sclerosis: histologic and biochemical features

**DOI:** 10.1186/s40478-021-01217-3

**Published:** 2021-06-28

**Authors:** Makoto Sainouchi, Yuya Hatano, Mari Tada, Tomohiko Ishihara, Shoichiro Ando, Taisuke Kato, Jun Tokunaga, Gaku Ito, Hiroaki Miyahara, Yasuko Toyoshima, Akio Yokoseki, Tetsutaro Ozawa, Kohei Akazawa, Osamu Onodera, Akiyoshi Kakita

**Affiliations:** 1 Departments of Pathology, Brain Research Institute, Niigata University, 1-757 Asahimachi, Chuo-ku, Niigata, 951-8585 Japan; 2 Departments of Neurology, Brain Research Institute, Niigata University, 1-757 Asahimachi, Chuo-ku, Niigata, 951-8585 Japan; 3 Department of System Pathology for Neurological Disorders, Brain Science Branch, Brain Research Institute, Niigata University, Niigata, Japan; 4 Department of Neurology, Uonuma Institute of Community Medicine, Niigata University Medical and Dental Hospital, 4132 Urasa, Minamiuonuma, Niigata 949-7302 Japan; 5 Department of Medical Informatics, Niigata University Medical and Dental Hospital, 1-754 Asahimachi, Chuo-ku, Niigata, 951-8520 Japan

## Correction to: acta neuropathol commun (2021) 9:106 https://doi.org/10.1186/s40478-021-01202-w

In the original publication [1] a contribution statement was accidentally removed during the publication process.

This statement is as followed:

- Makoto Sainouchi and Yuya Hatano contributed equally to this work

The original article has been updated. We apologize to the authors and readers for the inconvenience.
