# Supplementary material for: A novel splicing variant of ANXA11 in a patient with amyotrophic lateral sclerosis: histologic and biochemical features
Source: Acta Neuropathol Commun. 2021 Jun 7;9:106. doi: 10.1186/s40478-021-01202-w (PMC8186038; doi:10.1186/s40478-021-01202-w)

Additional file 1

**A novel splicing variant of ANXA11 in a patient with amyotrophic lateral sclerosis: histologic and biochemical features**

Makoto Sainouchi^1*^, Yuya Hatano^2*^, Mari Tada^1^, Tomohiko Ishihara^2^, Shoichiro Ando^2^, Taisuke Kato^3^, Jun Tokunaga^2^, Gaku Ito^2^, Hiroaki Miyahara^1^, Yasuko Toyoshima^1^, Akio Yokoseki^2^, Tetsutaro Ozawa^4^, Kohei Akazawa^5^, Osamu Onodera^2^, Akiyoshi Kakita^1^

^*^Makoto Sainouchi and Yuya Hatano contributed equally to this work.

Departments of ^1^Pathology, and ^2^Neurology, Brain Research Institute, Niigata University, 1-757 Asahimachi, Chuo-ku, Niigata 951-8585, Japan.

^3^Department of System Pathology for Neurological Disorders, Brain Science Branch, Brain Research Institute, Niigata University.

^4^Department of Neurology, Uonuma Institute of Community Medicine, Niigata University Medical and Dental Hospital, 4132 Urasa, Minamiuonuma, Niigata 949-7302, Japan.

^5^Department of Medical Informatics, Niigata University Medical and Dental Hospital, 1-754 Asahimachi, Chuo-ku, Niigata 951-8520, Japan.

Correspondence to: Mari Tada, MD, PhD^1^, or Tomohiko Ishihara, MD, PhD^2^

Department of Pathology^1^ and Neurology^2^, Brain Research Institute, Niigata University, 1-757 Asahimachi, Chuo-ku, Niigata 951-8585, Japan.

TEL: +81-25-2270636^1^; +81-25-2270666^2^

FAX: +81-25-2270817^1^; +81-25-2236646^2^

E-mail: tadamari@bri.niigata-u.ac.jp^1^; ishihara@bri.niigata-u.ac.jp^2^

Supplementary methods

Case presentation

Supplementary references

Supplementary table 1: Primary antibodies

Supplementary figure 1 and legend

**Supplementary methods**

**Neuropathologic analysis**

The brains and spinal cords were fixed with 10% buffered formalin, and multiple tissue blocks were embedded in paraffin. Histological examination was performed on 4-μm-thick sections using hematoxylin and eosin and Klüver-Barrera staining.

In addition, selected sections were immunostained with antibodies listed in **Supplementary table 1**, as described previously [11]. To test the specificity of the anti-annexin A11 antibody, we performed immunohistochemistry on paraffin-embedded tissue sections of the spinal cord and motor cortex of 3 individuals without any neurological disorders (3 men aged 47, 63, and 78 years) as healthy controls, and other patients with sporadic ALS cases devoid of known mutations (a man aged 63, and 2 women aged 71 and 74 years) as disease controls. Bound antibodies were visualized by the peroxidase-polymer-based method using a Histofine Simple Stain MAX-PO kit (Nichirei, Tokyo, Japan) with diaminobenzidine as the chromogen. Immunostained sections were counterstained with hematoxylin.

Double-label immunofluorescence was performed on sections of the spinal cord and motor cortex using antibodies against annexin A11 (1:50) with pS409/410 of TDP-43 (1:4000) or p62 (1:500). Secondary antibodies used were Alexa Fluor 488 goat anti-rabbit IgG (Molecular Probes, Eugene, OR, USA) and Alexa Fluor 568 goat anti-mouse IgG (Molecular Probes). The sections were treated with an Autofluorescence Eliminator Reagent (Millipore, Billerica, MA, USA), then mounted under glass coverslips using Vectashield mounting medium with 4,6-diamidino-2-phenylindole (DAPI) nuclear stain (Vector Laboratories, Burlingame, CA, USA). The sections were analyzed using a confocal laser scanning microscope (LSM700, Carl Zeiss, Oberkochen, Germany).

**Genetic analysis**

Genomic DNA was extracted from the fresh-frozen motor cortex using a QIAamp DNA Midi Kit (Qiagen, Hilden, Germany), and DNA quality checks were performed at the Agilent 2200 tape station (Agilent Technologies, Santa Clara, CA, USA). Exome analysis was performed by the outsourcing service provider (Takara Bio, Shiga, Japan) using an Illumina NovaSeq 6000. The number of unique and mapped reads was 1,512,756, the mean read depth of the target regions was 115.7, and 96.4% of the target regions showed at least 20-fold coverage.

These exome databases were interrogated for 39 ALS-related genes [1-5, 9] (including *TARDBP, OPTN, FUS, SOD1, TBK1, SQSTM1, MATR3, TUBA4A, NEK1, HNRNPA2B1, VCP, ELP3, SETX, HNRNPA1, CCNF, VAPB, C21orf2, CHCHD10, NEFH, ANG, DCTN1, CHMP2B, UBQLN2, FIG4, PFN1, ARHGEF28, EWSR1, TAF15, ANXA11, DAO, ERBB4, TIA1, GLE1, PRPH, ALS2, SPG11, SIGMAR1, KIF5A,* and *DNAJC7)* to select variants with a minor allele frequency (MAF) <0.001 in the genetic variation database (HGVD: http://www.hgvd.genome.med.kyoto-u.ac.jp/ and ExAC All/EAS : https://gnomad.broadinstitute.org/). *C9orf72* intron1 GGGGCC repeats were measured separately using a previously published method [7]. We confirmed each variant identified in exome analysis by Sanger analysis using an ABI PRISM 3130xl Genetic Analyzer (Thermo Fisher Scientific, Waltham, MA, USA).

**Splice site prediction**

Splice site prediction of the wild type and variant *ANXA11* was performed using Netgene 2 (http://www.cbs.dtu.dk/services/NetGene2/).

**Transcript investigation**

Transcript investigation was performed to confirm the effects of the splice site mutation of *ANXA11* we had identified on mRNA splicing. We extracted mRNA from frozen occipital lobe tissue of the patient using a mirVana™ miRNA isolation kit (Thermo Fisher Scientific), then produced complementary DNA (cDNA) by reverse transcriptase PCR using a SuperScript™ VILO™ kit (Thermo Fisher Scientific). cDNA was amplified using the primer pair: 5'-TGAAGCCTGCCTGATTGAGA-3' (forward) and 5'-CGGCCTGTCATTCTCTGGTA-3' (reverse) located at *ANXA11* exon 9 and exon 13. The numbering of exons was performed based on ANXA11-203 ([ENST00000422982.8](about:blank)) in the Ensembl Genome Browser (<https://asia.ensembl.org/index.html>.). The PCR products were isolated on a 1% agarose gel. The DNA fragments of interest were purified using a TA cloning kit (Thermo Fisher Scientific) and sequenced by the Sanger method using an ABI PRISM 3130xl Genetic Analyzer (Thermo Fisher Scientific). We also used the frozen occipital lobe tissue of 2 individuals who did not suffer from ALS (2 men aged 66, and 82 years) as healthy controls, and other patients with sporadic ALS cases devoid of known mutations (a man aged 78, and a woman aged 74 years) as diseased controls.

**Structural predictions**

Secondary structure prediction was performed using PSIPRED 4.0 (http://bioinf.cs.ucl.ac.uk/psipred/).

Motifs in normal and mutant annexin A11 proteins were searched using Pfam 34.0 ([http://pfam.xfam.org/](about:blank)) and HMMER v3.3.2 (http://hmmer.org/).

Hydrophobicity of the normal and mutant annexin A11 proteins was predicted and plotted [8] using ExPASy ([https://web.expasy.org/protscale/](about:blank)).

Structural disorder probabilities of normal and mutant annexin A11 proteins were predicted by the PrDOS (prediction of disordered protein regions from amino acid sequence server, <http://prdos.hgc.jp/cgi-bin/top.cgi>) [6]. A false positivity rate threshold of 5% was selected.

**Plasmid preparation and transfection**

The DNA fragment was synthesized using patient cDNA and restriction enzymes (BamHI and XhoI) recognizing the sequences of the PCR primers: 5'-ATCTCGAGATGAGCTACCCTGGCTATCC-3' (forward) and 5'-ATGGATCCTCAGTCATTGCCACCACAGA-3' (reverse). It was then inserted into the pEGFP-C3 vector (Clontech), and subcloned to express green fluorescent protein (GFP)-tagged ANXA11 in mammalian cells. We verified all constructs by DNA sequencing. Human embryonic kidney (HEK) 293T cells were grown and transfected with the use of Lipofectamine 3000 (Invitrogen, Thermo Fisher).

**Fractionation of annexin A11 by solubility and immunoblotting**

Annexin A11 was fractionated by solubility as described previously [10] with several modifications. Briefly, 24 h after transfection, ANXA11-GFP^WT^ and ANXA11-GFP^MT^ construct-transfected HEK293T cells in a 9.5 cm^2^ well were washed with PBS, then scraped into 300 µl of NP-40 lysis buffer (1% NP-40, 20 mM Tris-HCl, pH 7.4, 150 mM NaCl, 5 mM EDTA, 10% glycerol) containing protease inhibitors. After sonication and 30 min rotation at 4°C, the lysate was centrifuged at 15,800 x *g* for 20 min at 4°C. The supernatant was kept as the soluble fraction. The insoluble pellet was resuspended in 100 µl of urea-SDS buffer (the NP-40 lysis buffer with 8M urea, 3% SDS), then sonicated. The extract was then centrifuged at 15,800 x *g* for 20 min at room temperature and the supernatant was kept as the insoluble fraction.

Protein concentrations of soluble fractions were determined using a Protein Assay BCA Kit (Wako, Osaka, Japan), and then diluted to 1 µg/µl. The insoluble fractions were diluted in the same ratio as the corresponding soluble fractions. A total of 15 µl of fractions was loaded per well of a 10% polyacrylamide gel (Wako). Gels were transferred onto polyvinylidene difluoride (PVDF) membranes (Merck Millipore, Darmstadt, Germany), and stained with Ponceau S. For blocking, the PVDF membranes were incubated in TBS with 0.05% Tween 20 with 5% skim milk for 2 h. The blots were probed overnight at 4°C with primary antibody, then for one hour at room temperature with secondary antibodies. The primary antibodies used for immunoblotting are listed in **Supplementary table 1**. HRP-conjugated secondary antibodies (Polyclonal Mouse Anti-Goat Immunoglobulins, AP186P, Chemicon; Polyclonal Goat Anti-Mouse Immunoglobulins, P0447, Dako) were used, and the chemiluminescent reaction was performed using Immobilon Western Chemiluminescent HRP Substrate (Merck Millipore, Darmstadt, Germany). Actin was used as a loading control. The signal was visualized and analyzed by the Amersham Imager 680 (GE Healthcare, Chicago, IL, USA).

**Estimation of the intracellular localization of annexin A11**

Twenty-four hours after transfection, cells were fixed with 4% paraformaldehyde at room temperature for 20 min, and rinsed with PBS three times. The cells were counterstained with Hoechst 33342 (1:10000, H3570, Thermo Fisher Scientific) for 5 min, then observed for GFP- fluorescence using an immunofluorescence microscope (BZ-9000, KEYENCE, Osaka, Japan) and a confocal laser scanning microscope (LSM700). Consistent estimation of annexin A11 distribution patterns was confirmed. The numbers of each of the transfected cells showing annexin A11 cytoplasmic aggregation, or diffuse distribution in the nucleus and cytoplasm were counted, and the proportion of each type of cell is shown in Fig. 2f. An observer who was not informed of the mutation status performed manual cell counting 6 times each for normal or mutant annexin A11. In each experiment, we counted 100 cells in five or more images taken at x20 using BZ-9000. Statistical analyses were performed using a Mann-Whitney *U* test, and data are presented as mean ± SE. Differences at *p* <0.05 were considered statistically significant.

**Case presentation**

A 57-year-old man, who had no family history of neurologic disorders, developed left hand weakness and lower extremity stiffness. Three months later, the muscle weakness involved all extremities, and neurologic examination revealed diffuse hyperreflexia, spasticity of the lower extremity, spastic gait, and fasciculation in the upper extremities. Electromyography revealed extensive denervation in the upper and lower extremity muscles, masseter muscle, and tongue. He was diagnosed as having ALS and treated with riluzole. He developed dysphagia and dysarthria, and became wheelchair-bound 12 months after disease onset. Three months later, noninvasive positive pressure ventilation (NPPV) and tube feeding were introduced. His cognitive function was well preserved. He died of sudden cardiac arrest 19 months after disease onset.

**Supplementary references**

1. Al-Chalabi A, van den Berg LH, Veldink J (2017) Gene discovery in amyotrophic lateral sclerosis: implications for clinical management. Nat Rev Neurol 13:96-104
2. Bakkar N, Kovalik T, Lorenzini I, Spangler S, Lacoste A, Sponaugle K, et al (2018) Artificial intelligence in neurodegenerative disease research: use of IBM Watson to identify additional RNA-binding proteins altered in amyotrophic lateral sclerosis. Acta Neuropathol 135:227-247
3. Chia R, Chiò A, Traynor BJ (2018) Novel genes associated with amyotrophic lateral sclerosis: diagnostic and clinical implications. Lancet Neurol 17:94-102
4. Dols-Icardo O, García-Redondo A, Rojas-García R, Borrego-Hernández D, Illán-Gala I, Muñoz-Blanco JL, et al (2018) Analysis of known amyotrophic lateral sclerosis and frontotemporal dementia genes reveals a substantial genetic burden in patients manifesting both diseases not carrying the *C9orf72* expansion mutation. J Neurol Neurosurg Psychiatry 89:162-168
5. Farhan SMK, Howrigan DP, Abbott LE, Klim JR, Topp SD, Byrnes AE, et al (2019) Exome sequencing in amyotrophic lateral sclerosis implicates a novel gene, DNAJC7, encoding a heat-shock protein. Nat Neurosci 22:1966-1974
6. Ishida T, Kinoshita K (2007) PrDOS: prediction of disordered protein regions from amino acid sequence. Nucleic Acids Res 35:W460-464
7. Konno T, Shiga A, Tsujino A, Sugai A, Kato T, Kanai K, et al (2013) Japanese amyotrophic lateral sclerosis patients with GGGGCC hexanucleotide repeat expansion in C9ORF72. J Neurol Neurosurg Psychiatry 84:398-401
8. Kyte J, Doolittle RF (1982) A simple method for displaying the hydropathic character of a protein. J Mol Biol 157:105-132
9. Müller K, Brenner D, Weydt P, Meyer T, Grehl T, Petri S, et al (2018) Comprehensive analysis of the mutation spectrum in 301 German ALS families. J Neurol Neurosurg Psychiatry 89:817-827
10. Smith BN, Vance C, Scotter EL, Takes C, Wong CH, Topp S, et al (2015) Novel mutations support a role for Profilin 1 in the pathogenesis of ALS. Neurobiol Aging 36:1602.e17-27
11. Takeuchi R, Tada M, Shiga A, Toyoshima Y, Konno T, Sato T, et al (2016) Heterogeneity of cerebral TDP-43 pathology in sporadic amyotrophic lateral sclerosis: Evidence for clinico-pathologic subtypes. Acta Neuropathol Commun 4:61

**Supplementary table 1 Primary antibodies**

| **Antigen**  **(clone, catalog number)** | **Antibody species** | **Source** | **Dilution** | **Antigen retrieval** |
| --- | --- | --- | --- | --- |
| **Immunohistochemistry** |  |  |  |  |
| Annexin A11 (polyclonal, 10479-2-AP)  Phosphorylated TDP-43 (pS409/410) (11-9, TIP-PTD-M01) | Rabbit  Mouse | Proteintech, Rosemont, IL, USA  Cosmo Bio, Tokyo, Japan | 1:100  1:5000 | Heating  Heating |
| P62 lck ligand  (3/P62 LCK LIGAND, 610833) | Mouse | BD Biosciences, San Jose, CA, USA | 1:1000 | Heating |
| GFAP (polyclonal, PA5-16291) | Rabbit | Dako, Glostrup, Denmark | 1:1500 | None |
| Cystatin C (polyclonal, 80040-RP01) | Rabbit | Dako, Glostrup, Denmark | 1:4000 | None |
| **Western blot** |  |  |  |  |
| Annexin A11 (polyclonal, ab4121) | Goat | Abcam, Cambridge, UK | 1:2000 | None |
| GFP (polyclonal, 598) | Rabbit | MBL, Nagoya, Japan | 1:2000 | None |
| β-Actin (6D1, M177-3) | Mouse | MBL, Nagoya, Japan | 1:1000 | None |

**Supplementary figure 1**. **Expression of annexin A11 in CNS tissue of ALS patients with no *ANXA11* mutation and controls.**

(**a**-**f**) Sparse cytoplasmic dot-like staining in the anterior horn cells (**a**-**c**, ALS patients with no *ANXA11* mutation; **d**-**f**, controls). (**g**-**l**) A small number of cytoplasmic dot-like puncta in the pyramidal neurons of the frontal cortex (**g**-**i**, ALS patients with no *ANXA11* mutation; **j**-**l**, controls). Bar = 10 μm for all images. Annexin A11 immunohistochemistry.


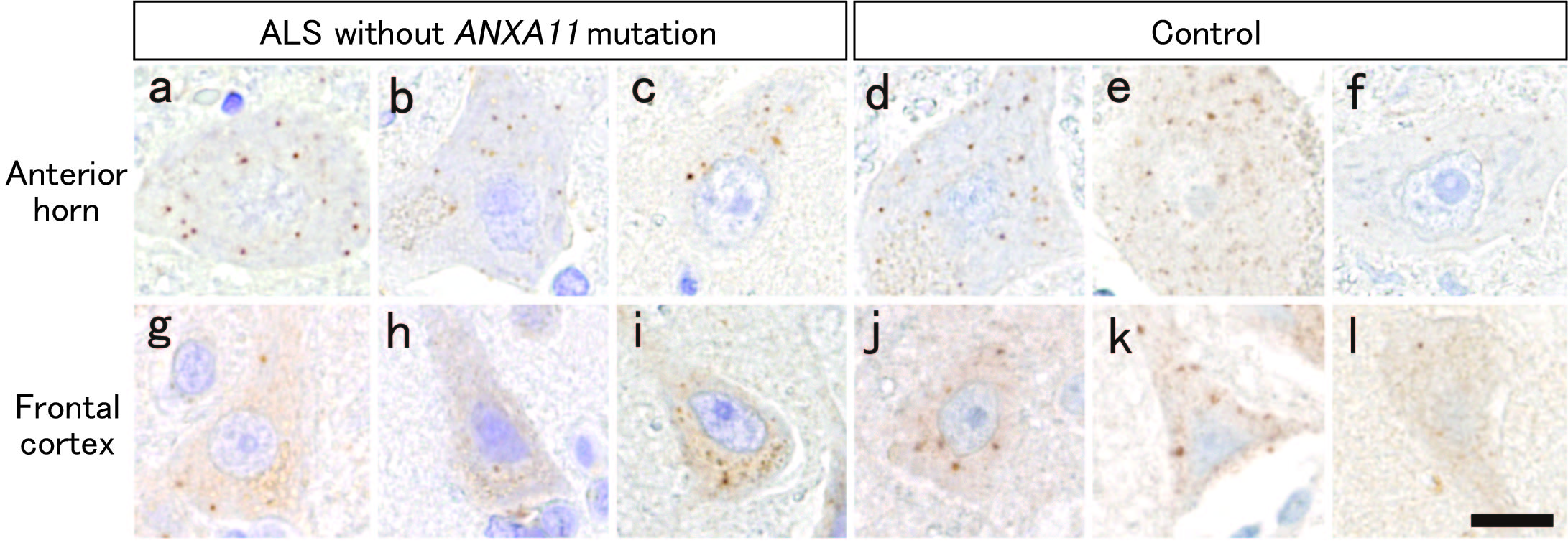

Supplement: Supplementary file 1 — Additional file 1. Details of methods and case presentation, a list of the primary antibodies used (Supplementary Table 1), and the representative images of annexin A11 immunohistochemistry in CNS tissue of controls (Supplementary Figure 1). [file 40478_2021_1202_MOESM1_ESM.docx]
